# Appointment of State Veterinarian for Pennsylvania

**Published:** 1896-01

**Authors:** 


### APPOINTMENT OF STATE VETERINARIAN FOR PENNSYLVANIA.

GOVERNOR HASTINGS, of Pennsylvania, on January 1st announced the appointment of Prof. Leonard Pearson as State Veterinarian at a salary of twenty-five hundred dollars per year. This appointment completes for the Keystone State the establishment of the State Veterinary Sanitary Board, and will inaugurate the work in Pennsylvania on a broad, conservative scale of dealing with infectious and contagious diseases among live stock. The appointment, after so long a wait, will be gratefully appreciated as a very acceptable New Year's gift by the veterinary profession of Pennsylvania. Well merited by virtue of peculiar abilities, training, and experience; broad-gauged and conservative, a thoroughly professional man, the State gains by this recognition one of the best-equipped men in our country, and Pennsylvania will now be found among the foremost commonwealths in preserving to our people her great live-stock interests and protecting them from the dangers that surround the meat- and milk-supply.

